# Hepatotoxicity of Clozapine : Case report and brief Review

**DOI:** 10.1192/j.eurpsy.2023.2137

**Published:** 2023-07-19

**Authors:** F. Askri, A. Aissa, S. Jedda, K. Mahfoudh, Y. Zgueb, U. Ouali

**Affiliations:** 1 Avicenna, razi psychiatric hospital, Manouba, Tunisia

## Abstract

**Introduction:**

Clozapine is an effective Atypical antipsychotic used in the treatment of resistant schizophrenia .However it can induce liver dysfunction from a simple transient asymptomatic cytolisis (30 to50 %) toa serious fulminant liver failure (0.001 %).

**Objectives:**

To show the heptotoxicity potential of Clozapine and adress the importance of monitoring the liver function tests in clozapine titration to prevent sever conditions

**Methods:**

A case report of a fifty-year old Tunisian male patient diagnosed with resistant schizophrenia who developed a hepatototoxicity under a low dose of clozapine within five days of treatment .

**Results:**

Mr F is a 50 year old patient diagnosed with schizophrenia in 2018 . He had received various antypical and typical antipsychotic treatments including ( Haloperidol , Risperidone , Amisulpride , Olanzapine ) at effective doses and minimal periods of six weeks . He had no history of systemic diseases or substance use disorder . He smokes 10 cigarettes a day . He had a history of hepatotoxicity on olanzapine. These medications have failed to resolve the persecutory delusion and auditory hallucinations , and the trial of clozapine was institued . Baseline examination and laboratory tests were normal . The previous antipsychotic medication was not continued and a dose of 25 mg clozapine was administred . A marking drowsiness was present in the fisrt days , so we decided to keep the same dose . Five days later , he had high levels of Liver function test (LFT) : Elevated aspartate ( 5 times above normal) and alanine aminotransferase levels (4 times above normal ) , white blood cell count and bilirubine levels were normal . He had no fever or jaundice . The abdominal examination showed a mild sensiblity in the right upper quadrant . Clozapine was immediatly discontinuated . 24 hours later LFT continued to escalate to 5 times greater then normal . Then it decreased continueosly

**Conclusions:**

Clozapine has a potential of hepatotoxicity even at lower dose . Screening liver function tests must be integrated in survey recommendations of clozapine treatment . Further researches must be conducted to understand the mechanism of this side effect in order to avoid sever conditions .

**Disclosure of Interest:**

None Declared

